# Prognostic Value of Circulating Fibrosis Biomarkers in Dilated Cardiomyopathy (DCM): Insights into Clinical Outcomes

**DOI:** 10.3390/biom14091137

**Published:** 2024-09-09

**Authors:** Elham Kayvanpour, Farbod Sedaghat-Hamedani, Daniel Tian Li, Tobias Miersch, Tanja Weis, Imo Hoefer, Norbert Frey, Benjamin Meder

**Affiliations:** 1 Department of Medicine III, University of Heidelberg, INF 410, 69120 Heidelberg, Germany; farbod.sedaghat-hamedani@med.uni-heidelberg.de (F.S.-H.); danieltianli@gmail.com (D.T.L.); tobias.miersch@gmx.net (T.M.); tanja.weis@med.uni-heidelberg.de (T.W.); norbert.frey@med.uni-heidelberg.de (N.F.); benjamin.meder@med.uni-heidelberg.de (B.M.); 2 DZHK (German Centre for Cardiovascular Research), 69120 Heidelberg, Germany; 3 Experimental Cardiology Laboratory, University Medical Center Utrecht, 3584 CX Utrecht, The Netherlands; i.hoefer@umcutrecht.nl; 4 Klaus Tschira Institute for Computational Cardiology, 69120 Heidelberg, Germany

**Keywords:** dilated cardiomyopathy, cardiac fibrosis, biomarkers, MMP-2, TIMP-1, GDF-15, OPN

## Abstract

Background: Dilated cardiomyopathy (DCM) involves myocardial remodeling, characterized by significant fibrosis and extracellular matrix expansion. These changes impair heart function, increasing the risk of heart failure and sudden cardiac death. This study investigates the prognostic value of circulating fibrosis biomarkers as a less invasive method in DCM patients. Methods: Plasma samples from 185 patients with confirmed DCM were analyzed to measure 13 circulating biomarkers using Luminex bead-based multiplex assays and ELISA. The prognostic value of these biomarkers was evaluated concerning heart failure-associated events and all-cause mortality. Results: Elevated MMP-2 levels (>1519.3 ng/mL) were linked to older age, higher diabetes prevalence, lower HDL, increased NT-proBNP and hs-TnT levels, and severe systolic dysfunction. High TIMP-1 levels (>124.9 ng/mL) correlated with elevated NT-proBNP, more atrial fibrillation, reduced exercise capacity, and larger right ventricles. Increased GDF-15 levels (>1213.9 ng/mL) were associated with older age, systemic inflammation, renal impairment, and poor exercise performance. Elevated OPN levels (>81.7 ng/mL) were linked to higher serum creatinine and NT-proBNP levels. Over a median follow-up of 32.4 months, higher levels of these biomarkers predicted worse outcomes, including increased risks of heart failure-related events and mortality. Conclusions: Circulating fibrosis biomarkers, particularly MMP-2, TIMP-1, GDF-15, and OPN, are valuable prognostic tools in DCM. They reflect the severity of myocardial remodeling and systemic disease burden, aiding in risk stratification and therapeutic intervention. Integrating these biomarkers into clinical practice could improve DCM management and patient prognosis.

## 1. Introduction

Myocardial remodeling in dilated cardiomyopathy (DCM) involves a multi-step process characterized by myocardial fibrosis and extracellular matrix expansion. These changes are direct consequences of neurohumoral pathway activation, cardiomyocyte apoptosis, and active cardiomyocyte-fibroblast signaling [1,2,3,4]. Fibrosis in DCM is a critical factor that leads to adverse clinical outcomes. The pathological accumulation of collagen and other extracellular matrix components not only affects the mechanical properties of the heart muscle but also alters its electrical properties, thereby increasing the risk of sudden cardiac death (SCD) and progression to heart failure [5]. The extent and pattern of fibrosis can vary significantly among DCM patients, highlighting the need for precise diagnostic tools to assess and quantify fibrosis in order to tailor treatment strategies effectively. Importantly, the presence of fibrosis also has significant implications for the efficacy of device therapies such as cardiac resynchronization therapy (CRT) and implantable defibrillators. As emphasized in recent studies, fibrosis can disrupt electrical pathways, leading to altered sensing and pacing thresholds in CRT, which may compromise the effectiveness of these therapies [6]. Therefore, assessing the extent of fibrosis is crucial not only for prognostication but also for optimizing device therapy in DCM patients.

To capture the extent of fibrosis and its impact, clinicians utilize various methods such as cardiac imaging tools, endomyocardial biopsy (EMB), and circulating biomarkers. Each method has its advantages and limitations. Cardiac magnetic resonance imaging (cMRI) with late gadolinium enhancement (LGE) is a non-invasive technique that can visualize myocardial fibrosis, providing detailed images of scar tissue and its distribution within the myocardium. However, cMRI has limitations in quantifying diffuse interstitial fibrosis accurately, which is a common feature in DCM [7]. EMB provides direct histopathological evidence of fibrosis. It allows for precise measurement of collagen volume fraction and detailed analysis of myocardial tissue architecture. Despite its invasive nature, which limits its widespread use, EMB remains particularly valuable in cases where non-invasive methods are inconclusive, or when there is a suspicion of specific myocardial conditions such as inflammation or infiltration [8]. Circulating biomarkers offer a less invasive means of assessing myocardial fibrosis. These biomarkers reflect the dynamic balance between collagen synthesis and degradation, providing insights into the ongoing fibrotic processes in the myocardium and can be measured through simple blood tests, making them attractive for routine clinical use. However, despite the potential of these biomarkers, their single-predictor value, reproducibility in clinical settings, and interdependence remain under investigation. Previous studies have shown mixed results regarding the prognostic significance of fibrosis biomarkers in DCM. Some studies have demonstrated strong associations between elevated biomarker levels and adverse outcomes, while others have reported inconsistent findings [9]. This variability may be due to differences in patient populations, study designs, and methods of biomarker measurement. Therefore, further research is needed to establish the clinical utility of these biomarkers and to determine whether they can reliably predict outcomes in DCM patients. This study aims to evaluate the prognostic value of 13 circulating fibrosis biomarkers in DCM patients, focusing on their ability to predict heart failure associated events and all-cause mortality. By identifying reliable biomarkers, this research seeks to enhance risk stratification and guide therapeutic interventions, ultimately improving patient outcomes in DCM.

## 2. Materials and Methods

### 2.1. Patient Enrollment, Follow-Up, and End Points

The study was conducted in accordance with the Declaration of Helsinki. The ethics committee of the University Hospital Heidelberg approved the inclusion and study of biomaterials and clinical data collected within the biobank initiative and all patients had given informed written consent. Plasma samples of 185 patients with clinically confirmed DCM were included. Relevant clinical and imaging data of patients from in-house records and registries were gathered including gender, body mass index (BMI), New York functional class (NYHA), cardiovascular risk factors, blood test results, 6-min walk test (6MWT), ECG, echocardiography, cardiac MRI, and cardiac device therapy. Two end points were defined: (i) heart failure associated events: composite end point of HF-related death, sudden cardiac death (SCD), aborted SCD (appropriate ICD shock, reported sustained ventricular tachycardia, or cardiopulmonary resuscitation), or cardiac transplantation and (ii) all-cause mortality. Events were either derived from longitudinal follow-up in the mentioned registries, in-house records, or by telephone contact. The composite end point for a patient was reached in case of the first event.

### 2.2. Biomarker Measurement

Plasma samples which had been collected at time of initial presentation were used to measure a group of 13 circulating biomarkers which have been proposed to reflect cardiac fibrosis [9]. Due to different sample dilution strategies, 3 different Luminex bead-based multiplex assays were designed to provide accurate quantification of the following target analytes: GDF-15, OPN, MMP-1, 3 & 8, Syndecan-1 & 4 (8-plex; sample dilution 2×; Galectin-3, MMP-2 & 9 (3-plex; sample dilution 50×). In addition, an extra assay was performed to quantify the target analyte TIMP-1 (single-plex; sample dilution 50×) since this target cannot be combined with the various MMP target analytes within the other target assays. In brief, samples were diluted in sample dilution buffer and 50 µL of the diluted sample was transferred to the designated well containing the proper amount of target specific beads. The plates were incubated for 2 h on a shaking platform at room temperature (RT) followed by washing of the plates using a 96-well plate washer. Next, plates were incubated for 1 h with target specific biotinylated antibodies followed by washing. A final incubation for 30 min was performed at RT with streptavidin-PE. All target analytes were quantified in duplo by reading the incubated plates on a Bio-Plex Multiplex immunoassay system (Bio-Rad, Hercules, CA, USA) in combination with Bio-Plex Manager 6.1.1. software (Bio-Rad). Plasma levels LAP were measured at the University Medical Center Utrecht, the Netherlands, using a semi-automated ELISA robot (Freedom EVO, Tecan, Männedorf, Switzerland). The intra-assay variation coefficient was 10 %. LAP was measured using a commercially available ELISA against human LAP according to the manufacturer’s instructions (RnDSystems). Soluble ST2 was also measured by ELISA following the manufacturer’s instructions (Critical Diagnostics).

### 2.3. Statistical Analysis

For each biomarker, a performance analysis was performed. The cut-off threshold was chosen at the maximum of the Youden index (Specificity + Sensitivity − 1), and the according specificity and sensitivity were calculated. Clinical and imaging characteristics were analyzed using standard descriptive statistics. Kaplan-Meier curves were used to show patients’ survival. All statistical tests were two-sided and *p* values < 0.05 were considered statistically significant. The statistical analyses were performed in R, version 3.2.1 or higher, using the packages “rms”, “Hmisc”, “ggplot2”, “survival”, “xtable”, “mice”, and “mitools”. 

## 3. Result

### 3.1. Patients’ Characteristics

In total, 185 DCM patients (mean age 55 ± 13.1 years; 63.2% male) were included in this study. 14.7% of the patients had a positive family history suggesting familial DCM. The detailed baseline characteristics of the study cohort are listed in Table 1. High-sensitive cardiac troponin T (hs-TnT) was only marginally increased (median 16.0 [9.0; 36.0] pg/mL), whereas NT-proBNP was strongly increased (median 1027.0 [278.0; 3588.0] ng/L) in the cohort. The mean LV-EF measured by echocardiography was 31.6 ± 12.5%. About 71% of patients were compensated at the time of presentation with a New York Heart Association (NYHA) functional class < III and could walk 395.5 ± 185.4 m in the six-minute walk test (6MWT).

### 3.2. Differences in Patient Characteristics by Biomarker Cut-Off Levels

**Figure 1:**
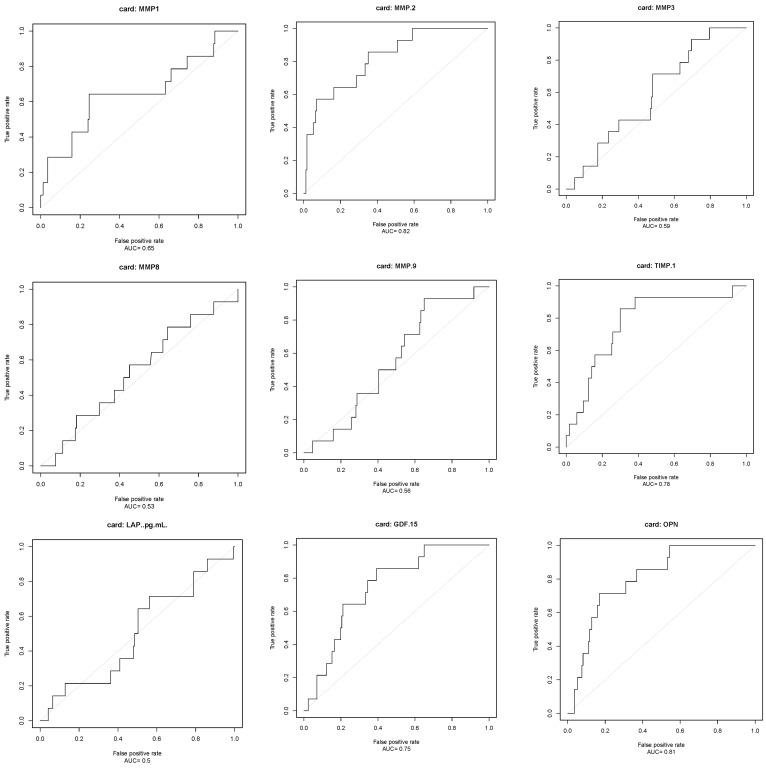

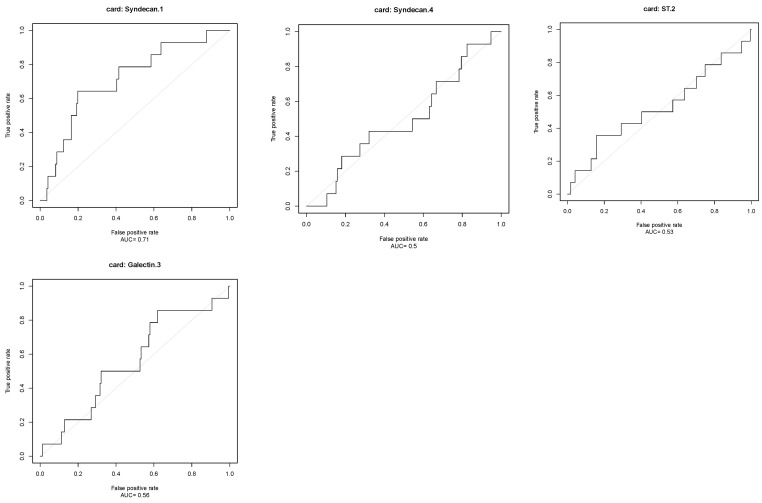
Circulating fibrosis biomarkers and their prognostic role in DCM: Receiver-operating characteristic (ROC) curves and corresponding area under the curve (AUC) statistics regarding composite endpoint.

13 circulating biomarkers were measured in patients’ blood samples as explained in the method section. Table 2 shows their median and interquartile range (IQR) values. Cut-off values for the biomarkers were determined and sensitivity, specificity, positive predictive, and negative predictive values for that certain cut-off point were calculated as described in the method (Table 3). Figure 1 shows receiver-operating characteristic (ROC) curves and corresponding area under the curve (AUC) statistics regarding composite endpoint.

Four of the 13 circulating biomarkers with the highest prognostic AUC values (MMP-2 with AUC 0.82, TIMP-1 with AUC 0.78, GDF-15 with AUC 0.75, and OPN with AUC 0.81) were selected for further analysis. The Supplementary Tables S1–S4 provide clinical and biochemical characteristics of patients divided by these biomarkers. Each biomarker categorized patients into two groups based on whether their levels were below or above the established cut-off values.

For MMP-2 levels (≤1519.3 ng/mL and >1519.3 ng/mL), significant differences were observed in age, diabetes prevalence, HDL levels, NT-proBNP, hs-TNT, dyspnea severity, and 6MWT results, indicating distinct disease trajectories and risk profiles. Patients with MMP-2 levels exceeding 1519.3 ng/mL were generally older (57.5 years vs. 53.5 years, *p* = 0.04) and had a higher prevalence of diabetes (23.4% vs. 9.7%, *p* = 0.04). They also exhibited lower HDL levels (40.4 mg/dL vs. 54.3 mg/dL, *p* < 0.01) and significantly elevated NT-proBNP levels (median: 2265.5 ng/L vs. 401.0 ng/L, *p* < 0.001). Similarly, hs-TNT levels were higher in this group (median: 33.0 pg/mL vs. 13.0 pg/mL, *p* = 0.001). Dyspnea severity was greater (NYHA III and IV, *p* < 0.001), and patients covered shorter distances in the 6MWT (227.5 m vs. 491.6 m, *p* = 0.01). Patients who were not receiving RAS inhibitors exhibited significantly higher levels of MMP-2 (*p* = 0.04). Echocardiography and cardiac MRI revealed significantly lower LV ejection fractions in patients with elevated MMP-2 levels (Echo: 25.5% vs. 34.3%, *p* < 0.001; MRI: 35.0% vs. 43.2%, *p* < 0.01), suggesting more severe systolic dysfunction. The LV end-systolic volume (LV-ESV) index was higher (93.2 mL/m^2^ vs. 68.2 mL/m^2^, *p* = 0.01), as was the LV end-diastolic volume (LV-EDV) index (135.6 mL/m^2^ vs. 113.4 mL/m^2^, *p* = 0.03), indicating greater ventricular dilation. The LV end-systolic diameter (LV-ESD) index was also significantly larger (26.3 mm/m^2^ vs. 23.0 mm/m^2^, *p* = 0.04), further supporting the presence of increased ventricular size and potential remodeling. While the LV end-diastolic diameter (LV-EDD) index and RV end-diastolic diameter (RV-EDD) index did not show statistically significant differences, the trends pointed to more pronounced ventricular enlargement in patients with higher MMP-2 levels. Additionally, the mitral annular plane systolic excursion (MAPSE) was significantly reduced (7.8 mm vs. 9.6 mm, *p* = 0.01), indicating impaired longitudinal LV function in these patients.

Patients with TIMP-1 levels exceeding 124.9 ng/mL also showed notable differences. NT-proBNP levels were significantly higher in this group (median: 2726.0 ng/L) compared to the lower TIMP-1 group (median: 710.0 ng/L, *p* < 0.001). Atrial fibrillation was more prevalent in patients with elevated TIMP-1 levels (27.6% vs. 13.1%, *p* = 0.04). In terms of functional capacity, patients with higher TIMP-1 levels covered significantly shorter distances in the six-minute walk test (223.7 m vs. 460.0 m, *p* = 0.05). Echocardiographic data revealed that left ventricular ejection fraction (LV-EF) was lower in the elevated TIMP-1 group (27.3%) compared to the lower TIMP-1 group (33.2%) (*p* = 0.01), suggesting more severe systolic dysfunction. Cardiac MRI data showed a larger right ventricular end-diastolic diameter (RV-EDD) index in the high TIMP-1 group (25.8 mm/m^2^ vs. 23.0 mm/m^2^, *p* = 0.04), indicating increased right ventricular size. Furthermore, the extent of late gadolinium enhancement (LGE), which reflects myocardial fibrosis, was higher in the elevated TIMP-1 group (5.9% vs. 4.0%, *p* = 0.05).

In the case of GDF-15, patients with levels above 1213.9 ng/mL were older on average (58.5 years vs. 52.5 years, *p* < 0.01), had a higher incidence of diabetes (25.4% vs. 6.2%, *p* < 0.01), and a higher BMI (28.7 kg/m^2^ vs. 26.7 kg/m^2^, *p* = 0.04). White blood cell count was higher in the elevated GDF-15 group (9.6/nL vs. 7.5/nL, *p* = 0.03), as were serum creatinine levels (1.4 mg/dL vs. 0.9 mg/dL, *p* < 0.001). NT-proBNP levels were significantly higher in patients with elevated GDF-15 (median: 2497.0 ng/L) compared to those with lower levels (median: 421.5 ng/L, *p* < 0.001). The elevated GDF-15 group also had a higher heart rate (90.6 beats/min vs. 68.9 beats/min, *p* < 0.01) and covered shorter distances in the 6MWT (247.8 m vs. 518.7 m, *p* < 0.01). The mean left ventricular ejection fraction was significantly lower in the higher GDF-15 group, indicating more severe systolic dysfunction. Furthermore, the mitral annular plane systolic excursion (MAPSE) was significantly lower in patients with higher GDF-15 levels (7.8 mm vs. 9.5 mm, *p* = 0.02), suggesting impaired longitudinal left ventricular function.

For OPN, patients with levels above 81.7 ng/mL had higher serum creatinine levels (mean: 1.5 mg/dL vs. 1.0 mg/dL, *p* < 0.001). Moreover, NT-proBNP levels were significantly elevated in the high OPN group (median: 3179.0 ng/L) compared to the lower OPN group (median: 956.0 ng/L, *p* = 0.05). Additionally, there were more females in patients with OPN levels above 81.7 ng/mL, reflecting a significant difference in gender distribution between the groups (*p* < 0.001).

### 3.3. Outcome Measures

**Figure 2:**
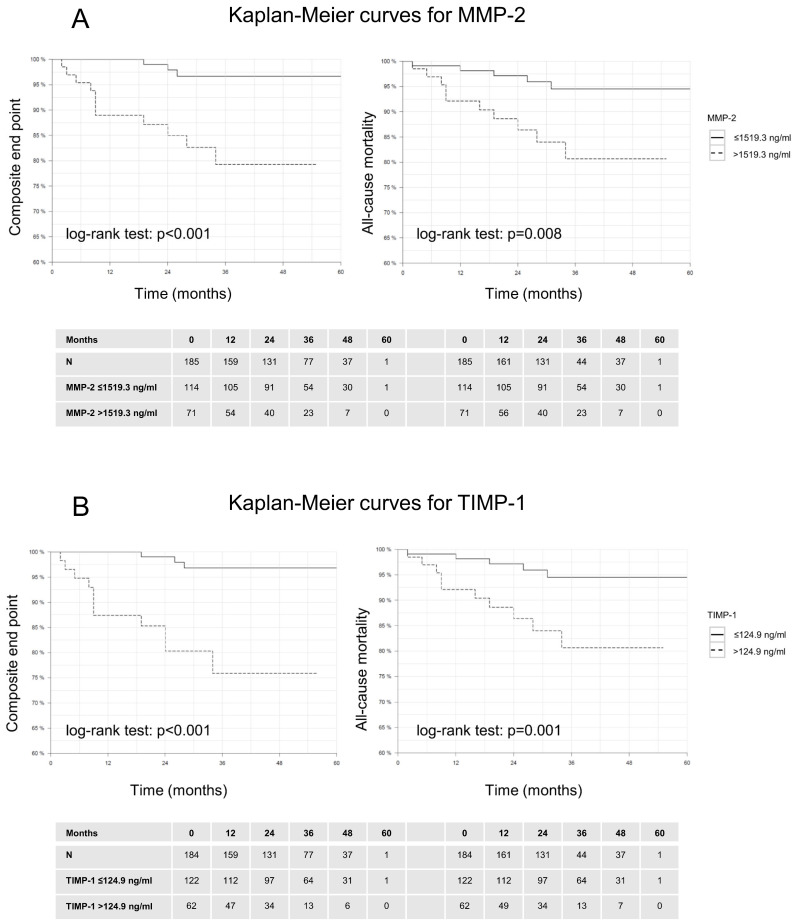
(**A**,**B**) Kaplan Meier survival estimates of the time to events depending on circulating levels of MMP-2 and TIMP-1. Patients with higher levels of these biomarkers show a significantly poorer survival rate in both endpoints.

**Figure 3:**
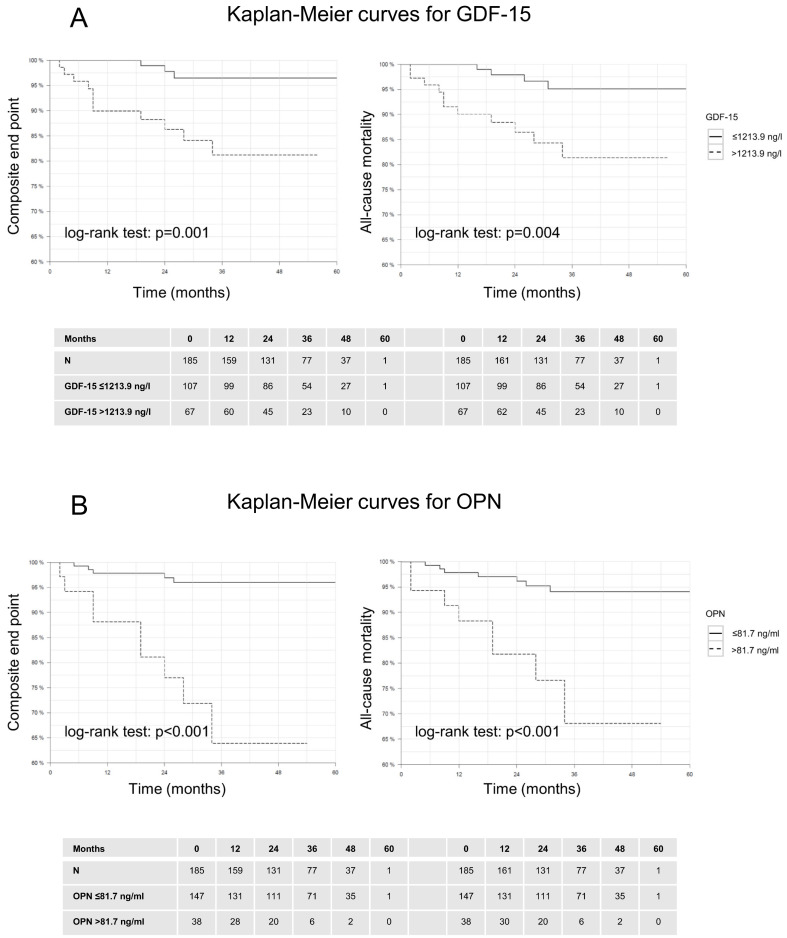
(**A**,**B**) Kaplan Meier survival estimates of the time to events depending on circulating levels of GDF-15 and OPN. Patients with higher levels of these biomarkers show a significantly poorer survival rate in both endpoints.

Patients were followed up for a median duration of 2.7 years (max. 5 years) representing 485.2 patient-years of follow-up. Altogether 14 patients reached the composite end point of HF associated events and 15 patients reached the end point all-cause mortality. We assessed the impact of the circulating biomarkers on patient outcomes using the defined cut-off values. Patients with elevated levels of MMP-2, TIMP-1, GDF-15, and OPN all demonstrated significantly poorer survival rates compared to those with lower levels of these biomarkers. The Kaplan-Meier survival estimates revealed substantial differences in both all-cause mortality and the composite endpoint, with the higher biomarker groups experiencing worse outcomes (Figure 2 and Figure 3).

## 4. Discussion

Many biohumoral parameters have been suggested as markers of cardiac fibrosis. These include molecules involved in collagen metabolism, turnover, and regulation, as well as chemokines that attract inflammatory cells to the site of injury. One well-documented pathway involves matrix metalloproteinases (MMPs) and their endogenous inhibitors, the tissue inhibitors of metalloproteinases (TIMPs) [10]. Several experimental and human studies have demonstrated an up-regulation of MMP-2 in end-stage heart failure (HF) [11,12,13,14,15]. However, data on circulating MMP-2 levels are inconclusive. While some studies have shown increased levels in congestive heart failure (CHF) patients, others have observed no significant changes [16,17]. Our study found that elevated MMP-2 levels (>1519.3 ng/mL) correlated with older age, higher diabetes prevalence, lower HDL levels, increased NT-proBNP and hs-TnT levels, and more severe systolic dysfunction. These findings suggest that high MMP-2 levels are indicative of more advanced disease. Similar to findings by George et al. we showed that higher serum MMP-2 levels were associated with poorer prognosis in DCM patients [18]. Furthermore, patients who did not receive RAS inhibitors exhibited significantly higher levels of MMP-2. This suggests a potential interaction between the absence of RAS inhibitor therapy and elevated MMP-2 levels in patients with DCM and emphasizes the importance of RAS inhibitors in such patients, not only for their hemodynamic effects but also for their role in modulating pathological remodeling processes.

TIMPs, particularly TIMP-1, have shown time-dependent changes in DCM. While Picard et al. showed a significant up-regulation of TIMP-1 in myocardial tissue of DCM patients in different disease stages, Polyakova et al. showed a non-significant elevation compared to controls and Li et al. showed a down-regulation in end-stage DCM patients [12,19,20]. Schwartzkopff et al. showed an elevated level of TIMP-1 in serum of patients with mild to moderate DCM [21]. In our study higher TIMP-1 levels (>124.9 ng/mL) were associated with higher NT-proBNP levels, a greater prevalence of atrial fibrillation, reduced exercise capacity, and larger right ventricular size. These associations underscore the potential of TIMP-1 as a biomarker for disease severity and prognosis in DCM, reflecting both myocardial fibrosis and systemic disease burden.

Growth differentiation factor (GDF)-15 has come under increasing scrutiny as a biomarker of HF. Its expression in cardiomyocytes can increase following exposure to ischemia or increased wall stress [22,23]. Lok et al. reported elevated level of serum GDF-15 in end-stage DCM the same as cardiac troponin and natriuretic peptides levels, whereas its mRNA and protein expression were hardly detectable in the heart tissue itself, suggesting that the myocardium is not the main source of GDF-15 in these patients [24]. The increased levels of the circulating GDF-15 decreased rapidly to near normal values within 1 month of left ventricular assist device (LVAD) support reflecting the peripheral effect of left ventricular unloading. The correlation between circulating GDF-15 and the amount of intracardiac fibrosis seems to improve with disease progress as shown by Lok et al. (R = 0.61, *p* = 0.01) [24]. Elevated GDF-15 levels (>1213.9 ng/mL) in our study were linked to older age, higher incidence of diabetes, systemic inflammation, renal impairment, and poorer exercise performance. The correlation between high GDF-15 levels and adverse outcomes aligns with its role in fibrosis and disease progression, suggesting that GDF-15 is a valuable prognostic marker in DCM.

Osteopontin (OPN) has been suggested essential for the differentiation and activation of myofibroblasts in response to the pro-fibrotic cytokine TGF-β1 during myocardial remodeling [25,26]. Furthermore, it inhibits MMPs involved in collagen degradation [27]. Almost undetectable in healthy myocardium, its intracardiac expression increases in failing hearts in correlation with cardiac fibrosis, insoluble collagen deposits, ventricular stiffness as well as decreasing LV-EF [28,29,30]. Elevated OPN levels (>81.7 ng/mL) in our cohort were associated with increased serum creatinine and NT-proBNP levels, reflecting renal dysfunction and a higher fibrotic burden. Its prognostic value for adverse outcomes was significant, supporting its role as a marker of disease severity.

LGE was not significantly different between groups with higher versus lower levels of these 4 biomarkers. These findings suggest that there may not be a direct correlation between LGE and the biomarkers in our cohort. However, further investigation into this potential relationship, particularly concerning arrhythmic risk and disease severity, remains of great interest. Future studies should explore this aspect more thoroughly to gain a comprehensive understanding of the prognostic role of these biomarkers in conjunction with imaging findings.

## 5. Conclusions

This study underscores the significant prognostic value of circulating fibrosis biomarkers in patients with dilated cardiomyopathy (DCM). The biomarkers MMP-2, TIMP-1, GDF-15, and OPN were particularly notable for their strong associations with adverse clinical outcomes, including heart failure events and all-cause mortality. Elevated levels of these biomarkers were linked to more severe myocardial remodeling, systemic inflammation, and impaired functional capacity, indicating their potential utility in enhancing risk stratification and guiding therapeutic interventions. By providing a non-invasive means to assess the severity of fibrosis and overall disease burden, these biomarkers can play a crucial role in personalizing treatment strategies and improving patient management, particularly when considered as an additive tool alongside conventional markers already used in patients with DCM and heart failure. Future research should focus on further validating these biomarkers and exploring the underlying biological mechanisms to fully integrate them into clinical practice for the benefit of DCM patients.

## Figures and Tables

**Table 1:** Baseline characteristics.

Characteristics	All Patients (Max. N = 185)
Age, mean (SD), years	55.0 (13.1)
Males, *n* (%)	117 (63.2)
BMI, mean (SD), kg/m^2^	27.6 (6.2)
Arterial hypertension, *n* (%)	122 (84.1)
Diabetes, *n* (%)	20 (14.3)
Tobacco smoking	
Current smoker, *n* (%)	26 (27.4)
Never smoker, (%)	40 (42.1)
Quit smoking, *n* (%)	29 (30.5)
Alcohol excess *, *n* (%)	0 (0.0)
Family history of CV disease, *n* (%)	19 (14.7)
Lipid profile	
Total cholesterol, mean (SD), mg/dL	189.0 (42.7)
High-density lipoprotein, mean (SD), mg/dL	48.2 (14.9)
Low-density lipoprotein, mean (SD), mg/dL	113.1 (30.0)
Triglyceride, mean (SD), mg/dL	174.1 (110.8)
White blood cell count, mean (SD),/nL	8.3 (3.5)
Hemoglobin, mean (SD), g/dL	13.7 (1.8)
Serum creatinine, mean (SD), mg/dL	1.1 (0.9)
NT-proBNP, median (IQR), ng/L	1027.0 [278.0; 3588.0]
hs-TNT, median (IQR), pg/mL	16.0 [9.0; 36.0]
Heart rate, mean (SD), beats/min	79.2 (19.5)
Left bundle-branch block, *n* (%)	5 (25.0)
Atrial fibrillation, *n* (%)	42 (22.8)
Blood pressure, mean (SD), mmHg	
Systolic	125.1 (16.9)
Diastolic	74.1 (14.3)
Dyspnoea, *n* (%)	
NYHA I	30 (54.5)
NYHA II	9 (16.4)
NYHA III	7 (12.7)
NYHA IV	9 (16.4)
6MWT, mean (SD), m	395.5 (185.4)
VO_2_ max, mean (SD), mL/(kg·min)	16.0 (6.1)
Medication at first visit	
Beta blocker	137 (75.3)
RAS inhibitor	140 (76.9)
Echocardiography	
LV ejection fraction, mean (SD)	31.6 (12.5)
Cardiac MRI data	
LV ejection fraction, mean (SD)	40.3 (13.2)
LV stroke volume, mean (SD), mL	92.5 (29.1)
LV-ESV index, mean (SD), mL/m^2^	76.9 (41.4)
LV-EDV index, mean (SD), mL/m^2^	121.1 (40.6)
LV-ESD index, mean (SD), mm/m^2^	24.1 (6.4)
LV-EDD index, mean (SD), mm/m^2^	30.5 (5.3)
LV mass index, mean (SD), g/m^2^	59.9 (19.3)
Septum wall thickness, mean (SD), mm	9.6 (2.2)
RV-EDD index, mean (SD), mm/m^2^	23.7 (4.3)
LA diameter, mean (SD), mm	39.5 (8.8)
MAPSE, mean (SD), mm	8.9 (3.2)
TAPSE, mean (SD), mm	18.6 (5.0)
Extent of late gadolinium enhancement, %, SD	4.5 (3.0)

* Defined as consistent intake of 4 or more units/d for men and 3 or more units/d for women. Abbreviations: CV: cardiovascular, 6MWT, six-minute walk test; ACE, angiotensin-converting enzyme; ARB, angiotensin II receptor blocker; BMI, body mass index; CV, cardiovascular; DCM, dilated cardiomyopathy; hs-TNT, high-sensitivity troponin T; IQR, interquartile range; LA, left atrium; LV, left ventricular; LV-EDD, left ventricular end diastolic diameter; LV-EDV, left ventricular end diastolic volume; LV-ESD, left ventricular end systolic diameter; LV-ESV, left ventricular end systolic volume; MAPSE, mitral annular plane systolic excursion; MRI, magnetic resonance imaging; *n*, number; NYHA, New York Heart Association; NT-proBNP, N-terminal prohormone of brain natriuretic peptide; SD, standard deviation; RV-EDD, right ventricular end diastolic diameter; TAPSE, tricuspid annular plane systolic excursion; VF, ventricular fibrillation.

**Table 2:** Circulating biomarkers of cardiac fibrosis at baseline.

Biomarker	All Patients (Max. N = 185)
MMP-1, median (IQR), ng/L	566.71 [328.2, 960.6]
MMP-2, median (IQR),ng/mL	1385.0 [1119.9, 1785.5]
MMP-3, median (IQR), ng/L	6617.4 [4417.1, 8957.4]
MMP-8, median (IQR), ng/L	3756.6 [1736.6, 7914.2]
MMP-9, median (IQR), ng/mL	116.8 [59.4, 226.7]
TIMP-1, median (IQR), ng/mL	104.5 [78.2, 144.0]
LAP, median (IQR), ng/L	10362.0 [6457.0, 15039.5]
GDF-15, median (IQR), ng/L	1049.0 [708.2, 2041.1]
OPN, median (IQR), ng/mL	52.8 [35.9, 75.2]
Syndecan-1, median (IQR), ng/L	4038.7 [2735.2, 5430.4]
Syndecan-4, median (IQR), ng/L	2198.09 [1508.0, 2919.7]
Soluble ST2, median (IQR), ng/mL	13.4 [10.1, 19.2]
Galectin-3, median (IQR), ng/mL	11.6 [9.3, 15.7]

MMP, Matrix metalloproteinase; TIMP-1, Tissue inhibitor of metalloproteinases-1; LAP, Latency-associated peptide; GDF-15, Growth differentiation factor-15; OPN, Osteopontin.

**Table 3:** Sensitivity and specificity of 4 biomarkers of cardiac fibrosis in predicting HF associated events in DCM.

Variable	Cut-Off	Sensitivity (%)	Specificity (%)	PPV	NPV
MMP-2	>1519.3 ng/mL	86	65	17	98
TIMP-1	>124.9 ng/mL	86	70	19	98
GDF-15	>1213.9 ng/L	86	61	15	98
OPN	>81.7 ng/mL	71	83	26	97

MMP-2, Matrix metalloproteinase 2; TIMP-1, Tissue inhibitor of metalloproteinases-1; GDF-15, Growth differentiation factor-15; OPN, Osteopontin; PPV, positive predictive value; NPV, negative predictive value.

## Data Availability

The raw data supporting the conclusions of this article will be made available by the authors on request.

## Supplementary Materials

The following supporting information can be downloaded at: https://www.mdpi.com/article/10.3390/biom14091137/s1, Table S1: Baseline characteristics based on MMP-2 cut-off value; Table S2: Baseline characteristics based on TIMP-1 cut-off value; Table S3: Baseline characteristics based on GDF-15 cut-off value; Table S4: Baseline characteristics based on OPN cut-off value.
