# Supplementary material for: Prognostic Value of Circulating Fibrosis Biomarkers in Dilated Cardiomyopathy (DCM): Insights into Clinical Outcomes
Source: Biomolecules. 2024 Sep 9;14(9):1137. doi: 10.3390/biom14091137 (PMC11430616; doi:10.3390/biom14091137)
Supplement: Supplementary file 1 [file biomolecules-14-01137-s001.zip › biomolecules-3182861-supplementary.pdf]

Table S1: Baseline characteristics based on MMP-2 cut-off value

Characteristics	MMP-2≤1519.3 ng/ml	MMP-2>1519.3 ng/ml	P value
Age, mean (SD), years	53.5 (12.2)	57.5 (14.1)	**0.04**
Males, n (%)	72 (63.2)	45 (63.4)	1.0
BMI, mean (SD), kg/m2	27.4 (5.9)	27.8 (6.6)	0.68
Arterial hypertension, n (%)	84 (87.5)	38 (77.6)	0.15
Diabetes, n (%)	9 (9.7)	11 (23.4)	**0.04**
Tobacco smoking			0.22
Current smoker, n (%)	14 (23.0)	12 (35.3)	
Never smoker, (%)	25 (41.0)	15 (44.1)	
Quit smoking, n (%)	22 (36.1)	7 (20.6)	
Alcohol excess*, n (%)	0 (0.0)	0 (0.0)	
Family history of CV disease, n (%)	12 (13.8)	7 (16.7)	0.79
Lipid profile			
Total cholesterol, mean (SD),mg/dl	196.0 (39.2)	178.7 (46.7)	0.23
High-density lipoprotein, mean (SD), mg/dl	54.3 (13.8)	40.4 (12.8)	**<.01**
Low-density lipoprotein, mean (SD), mg/dl	120.7 (31.0)	102.5 (26.0)	0.10
Triglyceride, mean (SD),mg/dl	151.7 (77.2)	196.5 (140.9)	0.51
White blood cell count, mean (SD), /nl	8.5 (3.9)	8.1 (2.8)	0.69
Hemoglobin, mean (SD), g/dl	13.5 (1.7)	14.2 (2.0)	0.19
Serum creatinine, mean (SD), mg/dl	1.1 (0.8)	1.2 (1.0)	0.46
NT-proBNP, median (IQR), ng/l	401.0 [138.0;1412.0]	2265.5 [941.8;13720.5]	**<.001**
hs-TNT, median (IQR), pg/ml	13.0 [5.8;27.5]	33.0 [14.0;73.0]	**0.001**
Heart rate, mean (SD), beats/min	70.6 (13.9)	87.1 (21.1)	0.05
Left bundle-branch block, n (%)	3 (33.3)	2 (18.8)	0.62
Atrial fibrillation, n (%)	28 (24.6)	14 (20.0)	0.59
Blood pressure, mean (SD), mmHg			
Systolic	130.7 (18.1)	120.5 (15.2)	0.19
Diastolic	79.0 (13.7)	70.0 (14.1)	0.17
Dyspnoea, n (%)			**<.001**
NYHA I	29 (74.4)	1 (6.3)	
NYHA II	6 (15.4)	3 (18.8)	
NYHA III	0 (0.0)	7 (43.8)	
NYHA IV	4 (10.3)	5 (31.3)	
6MWT, mean (SD), m	491.6 (84.8)	227.5 (202.6)	**0.01**
VO2 max, mean (SD), ml/(kg·min)	17.4 (5.7)	9.0 (0.0)	
Medication at first visit			
Beta blocker	91 (80.5)	46 (66.7)	0.06
RAS inhibitor	93 (82.3)	47 (68.1)	**0.04**

Table S1: Baseline characteristics (continued)

Characteristics	MMP-2≤1519.3 ng/ml	MMP-2>1519.3 ng/ml	P value
Echocardiography			
LV ejection fraction, mean (SD)	34.3 (12.0)	25.5 (11.5)	**<.001**
Cardiac MRI data			
LV ejection fraction, mean (SD)	43.2 (12.7)	35.0 (12.7)	**<.01**
LV stroke volume, mean (SD), ml	104.5 (19.7)	80.5 (34.7)	0.29
LV-ESV index, mean (SD), mL/m2	68.2 (36.6)	93.2 (45.6)	**0.01**
LV-EDV index, mean (SD), mL/m2	113.4 (36.0)	135.6 (45.3)	**0.03**
LV-ESD index, mean (SD), mm/m2	23.0 (5.7)	26.3 (7.2)	**0.04**
LV-EDD index, mean (SD), mm/m2	29.8 (4.5)	32.0 (6.4)	0.11
LV mass index, mean (SD), g/m2	58.4 (20.7)	62.6 (16.3)	0.31
Septum wall thickness, mean (SD), mm	9.8 (2.2)	9.3 (2.4)	0.31
RV-EDD index, mean (SD), mm/m2	29.8 (4.5)	32.0 (6.4)	0.11
LA diameter, mean (SD), mm	38.9 (8.4)	40.8 (9.5)	0.38
MAPSE, mean (SD), mm	9.6 (3.2)	7.8 (2.9)	**0.01**
TAPSE, mean (SD), mm	19.3 (5.2)	17.5 (4.4)	0.09
Extent of late gadolinium enhancement, %, SD	4.3 (3.0)	5.1 (3.3)	0.54

* Defined as consistent intake of 4 or more units/d for men and 3 or more units/d for women. Abbreviations: CV: cardiovascular, 6MWT, six-minute walk test; ACE, angiotensin-converting enzyme; ARB, angiotensin II receptor blocker; BMI, body mass index; CV, cardiovascular; DCM, dilated cardiomyopathy; hs-TNT, high-sensitivity troponin T; IQR, interquartile range; LA, left atrium; LV, left ventricular; LV-EDD, left ventricular end diastolic diameter; LV-EDV, left ventricular end diastolic volume; LV-ESD, left ventricular end systolic diameter; LV-ESV, left ventricular end systolic volume; MAPSE, mitral annular plane systolic excursion; MRI, magnetic resonance imaging; n, number; NYHA, New York Heart Association; NT-proBNP, N-terminal prohormone of brain natriuretic peptide; SD, standard deviation; RV-EDD, right ventricular end diastolic diameter; TAPSE, tricuspid annular plane systolic excursion; VF, ventricular fibrillation.

Table S2: Baseline characteristics based on TIMP-1 cut-off value

**Characteristics**	**TIMP-1≤124.9 ng/ml**	**TIMP>124.9 ng/ml**	**P value**
Age, mean (SD), years	55.1 (13.6)	54.8 (12.1)	0.88
Males, n (%)	77 (62.6)	40 (64.5)	0.87
BMI, mean (SD), kg/m2	27.8 (5.9)	27.3 (6.7)	0.62
Arterial hypertension, n (%)	89 (87.3)	33 (76.7)	0.14
Diabetes, n (%)	12 (12.1)	8 (19.5)	0.29
Tobacco smoking			0.52
Current smoker, n (%)	18 (28.6)	8 (25.0)	
Never smoker, (%)	24 (38.1)	16 (50.0)	
Quit smoking, n (%)	21 (33.3)	8 (25.0)	
Alcohol excess*, n (%)	0 (0.0)	0 (0.0)	
Family history of CV disease, n (%)	12 (13.0)	7 (18.9)	0.42
Lipid profile			
Total cholesterol, mean (SD),mg/dl	185.8 (41.9)	192.8 (44.5)	0.62
High-density lipoprotein, mean (SD), mg/dl	50.3 (15.4)	45.6 (14.4)	0.38
Low-density lipoprotein, mean (SD), mg/dl	113.9 (31.0)	111.9 (29.7)	0.86
Triglyceride, mean (SD),mg/dl	155.3 (52.8)	180.3 (126.6)	0.75
White blood cell count, mean (SD), /nl	7.9 (3.7)	8.9 (3.3)	0.32
Hemoglobin, mean (SD), g/dl	13.7 (1.5)	13.7 (2.2)	0.90
Serum creatinine, mean (SD), mg/dl	1.1 (0.8)	1.2 (1.0)	0.37
NT-proBNP, median (IQR), ng/l	710.0 [234.3;767.0]	2726.0 [1440.0;14073.0]	**<.001**
hs-TNT, median (IQR), pg/ml	14.0 [7.3;34.0]	24.0 [10.0;63.5]	0.06
Heart rate, mean (SD), beats/min	71.6 (14.9)	85.0 (21.1)	0.12
Left bundle-branch block, n (%)	2 (25.0)	3 (25.0)	1.0
Atrial fibrillation, n (%)	34 (27.6)	8 (13.1)	**0.04**
Blood pressure, mean (SD), mmHg			
Systolic	127.0 (18.7)	123.8 (16.4)	0.68
Diastolic	77.0 (16.1)	72.1 (13.4)	0.47
Dyspnoea, n (%)			0.07
NYHA I	24 (63.2)	6 (35.3)	
NYHA II	6 (15.8)	3 (17.6)	
NYHA III	2 (5.3)	5 (29.4)	
NYHA IV	6 (15.8)	3 (17.6)	
6MWT, mean (SD), m	460.0 (140.0)	223.7 (205.8)	0.05
VO2 max, mean (SD), ml/(kg·min)	16.3 (5.8)	15.5 (9.2)	0.90
Medication at first visit			
Beta blocker	96 (80)	41 (66.1)	0.06
RAS inhibitor	98 (81.7)	42 (67.7)	0.05

Table S2: Baseline characteristics (continued)

Characteristics	TIMP-1≤124.9 ng/ml	TIMP-1>124.9 ng/ml	P value
Echocardiography			
LV ejection fraction, mean (SD)	33.2 (12.8)	27.3 (10.8)	**0.01**
Cardiac MRI data			
LV ejection fraction, mean (SD)	41.3 (12.2)	37.6 (15.9)	0.32
LV stroke volume, mean (SD), ml	104.8 (17.1)	72.0 (37.0)	0.26
LV-ESV index, mean (SD), mL/m2	72.9 (39.5)	87.9 (45.7)	0.18
LV-EDV index, mean (SD), mL/m2	117.6 (38.7)	130.8 (44.9)	0.23
LV-ESD index, mean (SD), mm/m2	23.3 (5.2)	26.4 (8.8)	0.14
LV-EDD index, mean (SD), mm/m2	29.8 (4.6)	32.6 (6.7)	0.08
LV mass index, mean (SD), g/m2	59.3 (19.8)	61.3 (18.1)	0.66
Septum wall thickness, mean (SD), mm	9.7 (2.2)	9.5 (2.4)	0.78
RV-EDD index, mean (SD), mm/m2	23.0 (3.3)	25.8 (5.8)	**0.04**
LA diameter, mean (SD), mm	39.1 (7.8)	40.8 (11.4)	0.54
MAPSE, mean (SD), mm	9.2 (3.1)	8.3 (3.6)	0.32
TAPSE, mean (SD), mm	18.8 (4.9)	18.0 (5.2)	0.50
Extent of late gadolinium enhancement, %, SD	4.0 (2.5)	5.9 (4.1)	0.05

* Defined as consistent intake of 4 or more units/d for men and 3 or more units/d for women. Abbreviations: CV: cardiovascular, 6MWT, six-minute walk test; ACE, angiotensin-converting enzyme; ARB, angiotensin II receptor blocker; BMI, body mass index; CV, cardiovascular; DCM, dilated cardiomyopathy; hs-TNT, high-sensitivity troponin T; IQR, interquartile range; LA, left atrium; LV, left ventricular; LV-EDD, left ventricular end diastolic diameter; LV-EDV, left ventricular end diastolic volume; LV-ESD, left ventricular end systolic diameter; LV-ESV, left ventricular end systolic volume; MAPSE, mitral annular plane systolic excursion; MRI, magnetic resonance imaging; n, number; NYHA, New York Heart Association; NT-proBNP, N-terminal prohormone of brain natriuretic peptide; SD, standard deviation; RV-EDD, right ventricular end diastolic diameter; TAPSE, tricuspid annular plane systolic excursion; VF, ventricular fibrillation.

Table S3: Baseline characteristics based on GDF-15 cut-off value

Characteristics	GDF-15≤1213.9 ng/ml	GDF-15>213.9 ng/ml	P value
Age, mean (SD), years	52.5 (12.6)	58.5 (13.0)	**<.01**
Males, n (%)	64 (59.8)	53 (67.2)	0.28
BMI, mean (SD), kg/m2	26.7 (5.7)	28.7 (6.6)	**0.04**
Arterial hypertension, n (%)	70 (83.3)	52 (85.2)	0.82
Diabetes, n (%)	5 (6.2)	15 (25.4)	**<.01**
Tobacco smoking			0.81
Current smoker, n (%)	13 (25.0)	13 (30.2)	
Never smoker, (%)	22 (42.3)	18 (41.9)	
Quit smoking, n (%)	17 (32.7)	12 (27.9)	
Alcohol excess*, n (%)	0 (0.0)	0 (0.0)	
Family history of CV disease, n (%)	12 (15.8)	7 (13.2)	0.80
Lipid profile			
Total cholesterol, mean (SD),mg/dl	194.1 (42.8)	182.3 (43.0)	0.41
High-density lipoprotein, mean (SD), mg/dl	52.2 (15.0)	43.1 (13.6)	0.09
Low-density lipoprotein, mean (SD), mg/dl	120.7 (32.8)	102.5 (22.6)	0.1
Triglyceride, mean (SD),mg/dl	180.8 (71.7)	167.3 (147.6)	0.84
White blood cell count, mean (SD), /nl	7.5 (2.3)	9.6 (4.6)	**0.03**
Hemoglobin, mean (SD), g/dl	13.6 (1.5)	13.9 (2.2)	0.55
Serum creatinine, mean (SD), mg/dl	0.9 (0.2)	1.4 (1.3)	**<.001**
NT-proBNP, median (IQR), ng/l	421.5 [130.0;1362.3]	2497.0 [1120.0;9876.0]	**<.001**
hs-TNT, median (IQR), pg/ml	14.0 [5.0;32.0]	19.0 [10.0;50.5]	0.07
Heart rate, mean (SD), beats/min	68.9 (13.8)	90.6 (19.0)	**<.01**
Left bundle-branch block, n (%)	3 (30.0)	2 (20.0)	1.0
Atrial fibrillation, n (%)	21 (19.8)	21 (26.9)	0.29
Blood pressure, mean (SD), mmHg			
Systolic	126.3 (12.9)	123.8 (20.9)	0.75
Diastolic	74.8 (12.4)	73.3 (16.7)	0.82
Dyspnoea, n (%)			0.11
NYHA I	25 (64.1)	5 (31.3)	
NYHA II	5 (12.8)	4 (25.0)	
NYHA III	3 (7.7)	4 (25.0)	
NYHA IV	6 (15.4)	3 (18.8)	
6MWT, mean (SD), m	518.7 (71.2)	247.8 (172.0)	**<.01**
VO2 max, mean (SD), ml/(kg·min)	17.4 (5.7)	9.0 (0.0)	
Medication at first visit			
Beta blocker	79 (75.2)	58 (75.3)	1.0
RAS inhibitor	81 (77.1)	59 (76.6)	1.0

Table S3: Baseline characteristics (continued)

Characteristics	GDF-15≤1213.9 ng/ml	GDF-15>213.9 ng/ml	P value
Echocardiography			
LV ejection fraction, mean (SD)	33.7 (12.2)	28.5 (12.4)	**0.02**
Cardiac MRI data			
LV ejection fraction, mean (SD)	42.2 (13.4)	36.3 (12.0)	**0.04**
LV stroke volume, mean (SD), ml	104.8 (17.1)	72.0 (37.0)	0.26
LV-ESV index, mean (SD), mL/m2	71.7 (39.1)	87.2 (44.7)	0.12
LV-EDV index, mean (SD), mL/m2	116.1 (36.8)	131.0 (46.5)	0.15
LV-ESD index, mean (SD), mm/m2	23.4 (6.2)	25.7 (6.8)	0.15
LV-EDD index, mean (SD), mm/m2	30.3 (5.2)	31.0 (5.5)	0.58
LV mass index, mean (SD), g/m2	58.3 (20.5)	63.0 (16.4)	0.26
Septum wall thickness, mean (SD), mm	9.4 (2.2)	10.2 (2.2)	0.13
RV-EDD index, mean (SD), mm/m2	23.2 (3.7)	24.8 (5.2)	0.14
LA diameter, mean (SD), mm	38.4 (8.2)	42.0 (9.7)	0.11
MAPSE, mean (SD), mm	9.5 (3.2)	7.8 (3.0)	**0.02**
TAPSE, mean (SD), mm	19.3 (4.9)	17.3 (4.9)	0.08
Extent of late gadolinium enhancement, %, SD	4.3 (3.3)	5.1 (3.0)	0.44

[*] Defined as consistent intake of 4 or more units/d for men and 3 or more units/d for women. Abbreviations: CV: cardiovascular, 6MWT, six-minute walk test; ACE, angiotensin-converting enzyme; ARB, angiotensin II receptor blocker; BMI, body mass index; CV, cardiovascular; DCM, dilated cardiomyopathy; hs-TNT, high-sensitivity troponin T; IQR, interquartile range; LA, left atrium; LV, left ventricular; LV-EDD, left ventricular end diastolic diameter; LV-EDV, left ventricular end diastolic volume; LV-ESD, left ventricular end systolic diameter; LV-ESV, left ventricular end systolic volume; MAPSE, mitral annular plane systolic excursion; MRI, magnetic resonance imaging; n, number; NYHA, New York Heart Association; NT-proBNP, N-terminal prohormone of brain natriuretic peptide; SD, standard deviation; RV-EDD, right ventricular end diastolic diameter; TAPSE, tricuspid annular plane systolic excursion; VF, ventricular fibrillation.

Table S4: Baseline characteristics based on OPN cut-off value

**Characteristics**	**OPN≤81.7 ng/ml**	**OPN≤81.7 ng/ml**	**P value**
Age, mean (SD), years	54.4 (12.9)	57.5 (13.8)	0.19
Males, n (%)	95 (64.6)	22 (57.9)	**<.001**
BMI, mean (SD), kg/m2	27.6 (5.8)	27.8 (7.4)	0.87
Arterial hypertension, n (%)	97 (82.9)	25 (89.3)	0.57
Diabetes, n (%)	13 (11.4)	7 (26.9)	0.06
Tobacco smoking			0.61
Current smoker, n (%)	23 (28.8)	3 (20.0)	
Never smoker, (%)	32 (40.0)	8 (53.3)	
Quit smoking, n (%)	25 (31.3)	4 (26.7)	
Alcohol excess*, n (%)	0 (0.0)	0 (0.0)	
Family history of CV disease, n (%)	17 (15.9)	2 (9.1)	0.53
Lipid profile			
Total cholesterol, mean (SD),mg/dl	183.0 (38.2)	210.6 (53.3)	0.11
High-density lipoprotein, mean (SD), mg/dl	47.7 (15.5)	50.7 (12.8)	0.66
Low-density lipoprotein, mean (SD), mg/dl	109.0 (26.6)	130.3 (39.6)	0.12
Triglyceride, mean (SD),mg/dl	189.3 (115.1)	98.0 (43.8)	0.31
White blood cell count, mean (SD), /nl	8.4 (3.5)	8.0 (3.9)	0.74
Hemoglobin, mean (SD), g/dl	13.8 (1.6)	13.5 (2.6)	0.63
Serum creatinine, mean (SD), mg/dl	1.0 (0.6)	1.5 (1.5)	**<.001**
NT-proBNP, median (IQR), ng/l	956.0 [249.0;2337.5]	3179.0 [473.8;20832.5]	0.05
hs-TNT, median (IQR), pg/ml	15.0 [7.0;34.0]	29.5 [9.8;79.8]	0.25
Heart rate, mean (SD), beats/min	77.7 (19.5)	88.7 (20.4)	0.38
Left bundle-branch block, n (%)	5 (29.4)	0 (0.0)	0.25
Atrial fibrillation, n (%)	31 (21.2)	11 (28.9)	0.75
Blood pressure, mean (SD), mmHg			
Systolic	128.0 (15.7)	108.3 (16.1)	0.06
Diastolic	74.5 (15.1)	71.7 (10.4)	0.76
Dyspnoea, n (%)			0.93
NYHA I	29 (53.3)	6 (60.0)	
NYHA II	8 (17.8)	1 (10.0)	
NYHA III	6 (13.3)	1 (10.0)	
NYHA IV	7 (15.6)	2 (20.0)	
6MWT, mean (SD), m	434.5 (140.2)	6 (0.0)	
VO2 max, mean (SD), ml/(kg·min)	14.8 (6.0)	22.0 (0.0)	
Medication at first visit			
Beta blocker	111 (77.1)	26 (68.4)	0.4
RAS inhibitor	113 (78.5)	27 (71.1)	0.5

Table S4: Baseline characteristics (continued)

Characteristics	OPN≤81.7 ng/ml	OPN≤81.7 ng/ml	P value
Echocardiography			
LV ejection fraction, mean (SD)	31.4 (12.8)	32.4 (11.1)	0.72
Cardiac MRI data			
LV ejection fraction, mean (SD)	39.9 (13.4)	42.9 (12.1)	0.44
LV-ESV index, mean (SD), mL/m2	78.0 (42.8)	69.0 (31.8)	0.41
LV-EDV index, mean (SD), mL/m2	122.5 (41.4)	112.0 (34.8)	0.38
LV-ESD index, mean (SD), mm/m2	24.1 (6.1)	24.6 (8.7)	0.86
LV-EDD index, mean (SD), mm/m2	30.6 (5.0)	30.2 (7.5)	0.87
LV mass index, mean (SD), g/m2	60.6 (19.7)	54.9 (15.7)	0.30
Septum wall thickness, mean (SD), mm	9.6 (2.2)	9.8 (2.8)	0.78
RV-EDD index, mean (SD), mm/m2	23.6 (4.1)	24.4 (5.7)	0.70
LA diameter, mean (SD), mm	39.6 (7.9)	39.3 (13.2)	0.94
MAPSE, mean (SD), mm	8.9 (3.2)	9.3 (3.4)	0.68
TAPSE, mean (SD), mm	18.5 (4.8)	19.1 (6.0)	0.77
Extent of late gadolinium enhancement, %, SD	4.7 (3.2)	3.5 (2.1)	0.33

* Defined as consistent intake of 4 or more units/d for men and 3 or more units/d for women. Abbreviations: CV: cardiovascular, 6MWT, six-minute walk test; ACE, angiotensin-converting enzyme; ARB, angiotensin II receptor blocker; BMI, body mass index; CV, cardiovascular; DCM, dilated cardiomyopathy; hs-TNT, high-sensitivity troponin T; IQR, interquartile range; LA, left atrium; LV, left ventricular; LV-EDD, left ventricular end diastolic diameter; LV-EDV, left ventricular end diastolic volume; LV-ESD, left ventricular end systolic diameter; LV-ESV, left ventricular end systolic volume; MAPSE, mitral annular plane systolic excursion; MRI, magnetic resonance imaging; n, number; NYHA, New York Heart Association; NT-proBNP, N-terminal prohormone of brain natriuretic peptide; SD, standard deviation; RV-EDD, right ventricular end diastolic diameter; TAPSE, tricuspid annular plane systolic excursion; VF, ventricular fibrillation.
